# Sildenafil Attenuates Inflammation and Oxidative Stress in Pelvic Ganglia Neurons after Bilateral Cavernosal Nerve Damage

**DOI:** 10.3390/ijms151017204

**Published:** 2014-09-26

**Authors:** Leah A. Garcia, Su M. Hlaing, Richard A. Gutierrez, Maria D. Sanchez, Istvan Kovanecz, Jorge N. Artaza, Monica G. Ferrini

**Affiliations:** 1 Division of Endocrinology, Metabolism and Molecular Medicine, Department of Internal Medicine, Charles R. Drew University of Medicine and Science, 1731 East 120th Street, Los Angeles, CA 90059, USA; E-Mails: lgarcia@ddchemco.com (L.A.G.); suhlaing@cdrewu.edu (S.M.H.); joartaza@ucla.edu (J.N.A.); 2 Department of Health and Life Science, College of Science and Health, Charles R. Drew University of Medicine and Science, Los Angeles, CA 90059, USA; E-Mails: RichardGutierrez@cdrewu.edu (R.A.G.); MariaSanchez@cdrewu.edu (M.D.S.); 3 Los Angeles Biomedical Research Institute, Harbor-University of California Los Angeles, Torrance, CA 90502, USA; E-Mail: ikovanecz@labiomed.org; 4 Department of Urology, David Geffen School of Medicine, University of California Los Angeles, Los Angeles, CA 90095, USA; 5 Department of Medicine, David Geffen School of Medicine, University of California Los Angeles, Los Angeles, CA 90095, USA

**Keywords:** sildenafil, cytokines, inducible nitric oxide synthase, NADPH oxidase, Heme oxygenase 1, bilateral cavernosal nerve damage

## Abstract

Erectile dysfunction is a common complication for patients undergoing surgeries for prostate, bladder, and colorectal cancers, due to damage of the nerves associated with the major pelvic ganglia (MPG). Functional re-innervation of target organs depends on the capacity of the neurons to survive and switch towards a regenerative phenotype. PDE5 inhibitors (PDE5i) have been successfully used in promoting the recovery of erectile function after cavernosal nerve damage (BCNR) by up-regulating the expression of neurotrophic factors in MPG. However, little is known about the effects of PDE5i on markers of neuronal damage and oxidative stress after BCNR. This study aimed to investigate the changes in gene and protein expression profiles of inflammatory, anti-inflammatory cytokines and oxidative stress related-pathways in MPG neurons after BCNR and subsequent treatment with sildenafil. Our results showed that BCNR in Fisher-344 rats promoted up-regulation of cytokines (interleukin- 1 (IL-1) β, IL-6, IL-10, transforming growth factor β 1 (TGFβ1), and oxidative stress factors (Nicotinamide adenine dinucleotide phosphate (NADPH) oxidase, Myeloperoxidase (MPO), inducible nitric oxide synthase (iNOS), TNF receptor superfamily member 5 (CD40) that were normalized by sildenafil treatment given in the drinking water. In summary, PDE5i can attenuate the production of damaging factors and can up-regulate the expression of beneficial factors in the MPG that may ameliorate neuropathic pain, promote neuroprotection, and favor nerve regeneration.

## 1. Introduction

Radical prostatectomy (RP) is currently the method of choice for the treatment of early stage prostate cancer. However, the most common complication of this procedure is the loss of adequate erectile function due to the damage or manipulation of the cavernosal nerves, which run across the prostate [1,2]. Damage of the cavernosal nerves causes changes in oxidative stress in the corpora cavernosa and a decrease of the neuronal nitric oxide synthase (nNOS) fibers in the dorsal and intracavernosal nerves [3], which in term causes apoptosis of the cavernosal smooth muscle cells and a change in the smooth muscle collagen ratio due to an increase in fibrosis, thus, negatively affecting erectile function [4].

The return of potency after surgical injury of the cavernosal nerves partially depends on axon regeneration in the remaining neural tissue by increasing the number of nNOS positive fibers in the corpora cavernosa [5,6] or by prophylactics pharmacological therapies aimed to reduce oxidative stress [7,8] or preserve the smooth muscle and reduce fibrosis in the penile corpora cavernosa. However, these palliative therapies cannot be sustained if nerve regeneration does not occur.

The process of regeneration and functional recovery of peripheral nerves is usually slow and often incomplete. Peripheral nerve regeneration is highly modulated by several factors, such as neurotrophic factors, extracellular matrix, and cellular components of the nervous system [9,10]. In addition, surgical trauma causes an inflammatory response and oxidative stress around the degenerating axons, which may cause neuronal damage due to retrograde reaction and chromatolysis [9].

Inflammatory cytokines including interleukin-1 (IL-1), IL-3, IL-6, and Tumor Necrosis Factor-α (TNFα), play an important role in the inflammatory process and are increased in response to tissue injury in the acute phase of surgical trauma [11]. Plasmatic levels of IL-6, IL-10, and other cytokines are significantly increased in patients immediately after or 24 h after radical prostatectomy [12,13]. In the nervous system, IL-6 plays a role in neuroprotection and in the modulation of pain [14,15]. Oxidative stress also plays a role in the induction of inflammatory cytokines [16]. Nicotinamide adenine dinucleotide phosphate (NADPH) oxidase, a major producer of ROS, increases superoxide and enhances the expression of inducible nitric oxide synthase (iNOS), as well as secretion of cytokine interleukin-1 (IL-1) β in microglial cells [17]. Moreover, iNOS expression can be induced by lipopolysaccharide and other inflammatory cytokines, often leading to high and persistent increases in nitric oxide (NO) [18]. Uncontrolled NO production can lead to tissue injury and damage to cellular constituents through the generation of reactive nitrogen species, such as peroxynitrite, which is involved in the nitration of proteins and lipids that prevents their normal functioning [19]. Furthermore, iNOS plays a pleiotropic effect since it is postulated to be involved in neurotoxicity and cell death in hypothalamic regions associated with reproduction during senescence [20], stroke [21,22], and nervous system damage [23]. It also acts as an anti-fibrotic agent counteracting fibrosis of the penile corpora cavernosa in aging, or injury, in the aged media of the penile arteries, and the tunica albuginea, protecting the erectile tissue. [24].

In previous studies, we have demonstrated that the use of PDE5 inhibitors, after bilateral cavernosal nerve resection (BCNR), preserves penile corporal smooth muscle and ameliorates fibrotic degeneration by down-regulating genes related to fibrosis and up-regulating genes related to smooth muscle preservation [25]. We have also observed that at the level of the pelvic ganglia, Sildenafil (SIL) exerts a neuroprotective effect by activating neurotrophic factors involved in neuronal survival and regeneration [26]. However, little is known of the role of NO/cyclic guanosine monophosphate (cGMP) pathways on cytokines and oxidative stress modulation after nerve resection in autonomic neurons.

This manuscript investigates the changes in oxidative stress and cytokines related-pathways in pelvic ganglia neurons after BCNR and the subsequent treatment with sildenafil in order to gain a further understanding of the role of PDE5 inhibitors on rehabilitation of the erectile function following radical prostatectomy.

## 2. Results

### 2.1. Bilateral Cavernosal Nerve Resection (BCNR) and Sildenafil Treatment Promotes Changes in the Gene Expression of Pro-Inflammatory and Anti-Inflammatory Cytokines

Changes in the gene expression profile of cytokines after seven days BCNR were studied by PCR array. Table 1 shows the list of cytokines that were up-regulated by BCNR. An increased expression of mRNA levels of pro-inflammatory cytokines, such as lL-1β, IL-6, IL-10 and its receptor IL-10Rα subunit, transforming growth factor beta 1 (TGFβ1), and TNF receptor superfamily member 5 (CD40) were observed. The treatment with SIL for seven days partially attenuated the expression of these cytokines, although the level of IL-6 and IL-10 still remained elevated.

**Table 1:** Changes in the gene expression profile of cytokines after bilateral cavernosal nerve resection (BCNR) and treatment with Sildenafil (SIL) determined by PCR array.

Symbol	Description	Sham *vs.* BCNR	BCNR *vs.* BCNR + SIL
CD40	CD40 molecule, TNF receptor superfamily member 5	3.68	−1.41
GMFγ	Glia maturation factor, γ	2.44	−1.16
IL-10	Interleukin 10	2.18	2.08
IL10Rα	Interleukin 10 receptor, α	4.03	−1.87
IL-1β	Interleukin 1 β	5.81	−1.18
IL-6	Interleukin 6	3.06	2.14
TGFβ1	Transforming growth factor, β 1	2.86	−1.35

**Figure 1:**
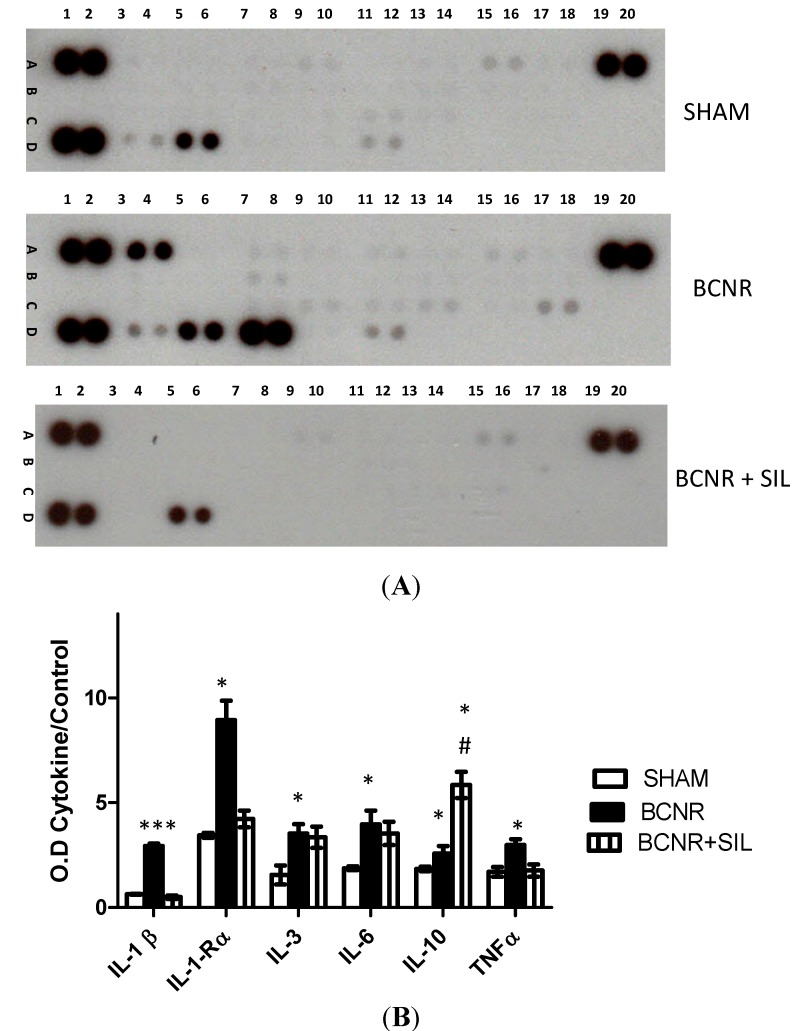
Cytokine expression is regulated by BCNR and SIL treatment. Protein extracts were incubated with a cocktail of biotinylated detection antibodies and then incubated in a membrane array containing 29 different anti-cytokine antibodies, in duplicates, followed by streptavidin peroxidase and chemoluminescence. The densitometric signal produced by each spot run in duplicate was proportional to the amount of cytokine bound as determined by densitometry analysis. (**A**) Representative membranes for Sham, BCNR and BCNR+SIL; (**B**) Densitometry analysis of cytokines * *p* < 0.05; *** *p* < 0.001 with respect to sham. # *p* < 0.05 with respect to BCNR.

To corroborate these findings at the protein level, a proteome array analysis was carried out, studying 29 different types of cytokines and chemokines. Figure 1 shows examples of representative membranes from each experimental group with its corresponding densitometric analysis. In agreement with our PCR array results, a significant up-regulation of several cytokines such as IL-1β, IL R-1α, IL-3, IL-10, IL-6, and TNFα was observed after BCNR. SIL treatment attenuated the increase of cytokine expression by down-regulating the expression of IL-1β, IL-R-1α, and TNFα. However, SIL treatment did not revert back the levels of IL-3, IL-6, and IL-10.

**Figure 2:**
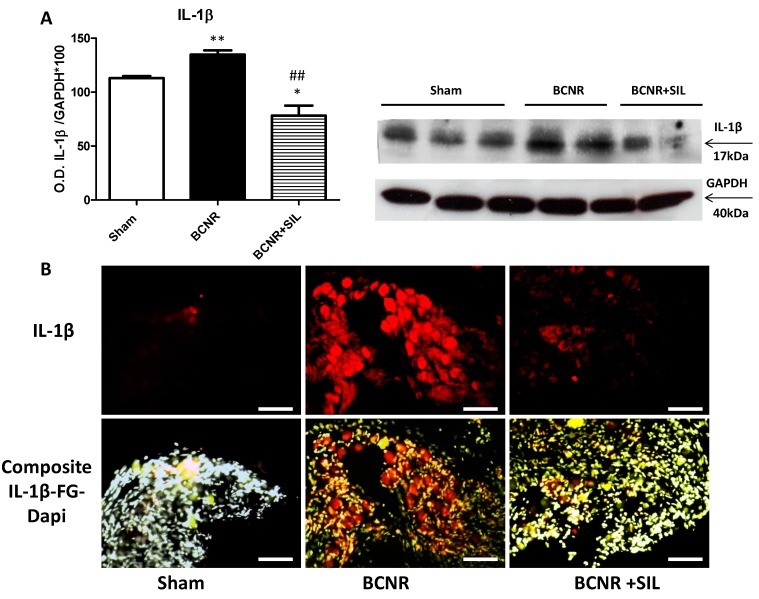
Sildenafil down regulates the expression of inflammatory cytokine IL-1β in the pelvic ganglia after BCNR. (**A**) Protein extracts were subjected to Western blot analysis for IL-1β and normalized by GAPDH with the correspondent densitometric analysis. * *p* < 0.05; ** *p* < 0.01 respect to sham. ## *p* < 0.01 respect to BCNR; (**B**) Frozen pelvic ganglia sections were immunostained with a polyclonal antibody against IL-1β followed by a biotinylated secondary antibody and streptavidin conjugated with Texas red. Red: IL-1β, Yellow: FG. White: DAPI. Magnification 100×; Bar=100 µm.

In order to confirm the findings obtained by both PCR and proteome array and to determine whether the expression of interleukins was localized in neurons or glial cells after BCNR, we studied the expression of IL-1β by Western blot and immunofluorescence respectively. Figure 2 shows that the expression of IL-1β was up-regulated by BCNR and down-regulated by SIL treatment. This was also corroborated by immunofluorescence and co-localization with fluorogold (FG). Interestingly, the expression of IL-1β was localized in neurons with or without FG positive staining, indicating that it was not only the axotomized neurons that were impacted by BCNR.

**Figure 3:**
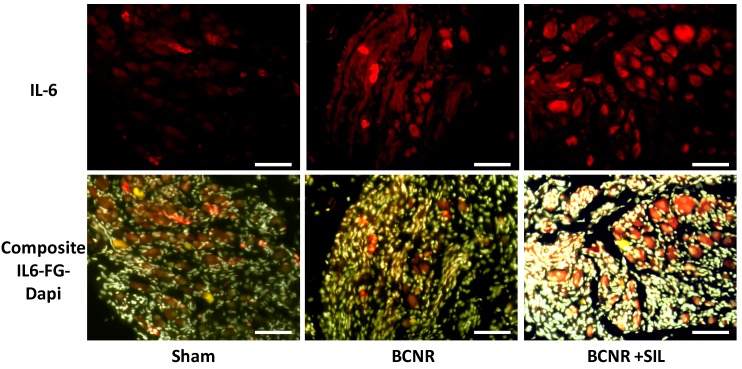
Sildenafil up-regulates the expression of IL-6 in the pelvic ganglia after BCNR. Frozen pelvic ganglia sections were immunostained with a polyclonal antibody against IL-6 followed by a biotinylated secondary antibody and streptavidin conjugated with Texas red. Red: IL-6, Yellow: FG. White: DAPI. Magnification 100×; Bar = 100 µm.

In order to corroborate that the levels of IL-6 were elevated after BCNR and remained up-regulated after SIL treatment, the expression of IL-6 was evaluated by immunofluorescence. Figure 3 shows that the expression of IL-6 in sham animals was circumscribed to a few non-FG positive cells. After BCNR, the IL-6 expression shifted to a few FG-positive cells and its expression was up-regulated by SIL in positive or negative FG cells.

### 2.2. Changes in Oxidative Stress Markers after BCNR and Sildenafil (SIL) Treatment

The impact of nerve damage and subsequent treatment with SIL on the expression of oxidative stress markers in the pelvic ganglia were evaluated by an RT-PCR array. A total of 54 genes were analyzed, but only 6 significantly changed after seven days of BCNR. Table 2 shows the list of oxidative stress-related genes. An up-regulation of the expression of glutathione peroxidase 1 and 7 (GPX1, GPX 7), Nitric oxide synthase 2 (iNOS) and subunits of the NADPH oxidase such as Neutrophil cytosolic factor 2 (ncf2), NADPH activator 1 (NOXA 1) and p-22-hox were observed after 7 days of BCNR in comparison to sham operated animals. The treatment with Sildenafil for 7 days after BCNR produced a down regulation of mRNA expression of the above genes. In addition, a reduced expression of myeloperoxidase (MPO) was observed after SIL treatment. No changes in the expression of endothelial Nitric oxide synthase (eNOS) were observed after BCNR or SIL treatment.

**Figure 4:**
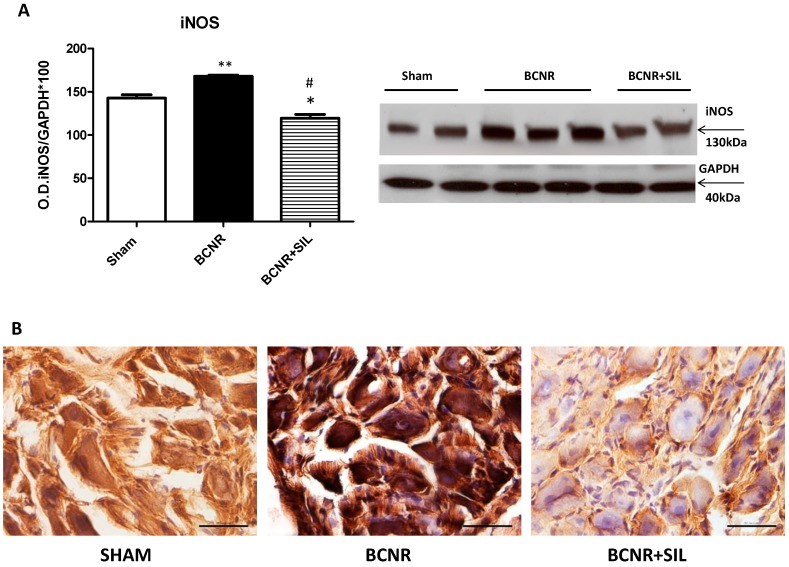
Inducible nitric oxide synthase (iNOS) expression is down regulated by Sildenafiltreatment after BCNR. (**A**) Total protein extracts of Sham, BCNR and BCNR+SIL were subjected to Western blot studies for iNOS and normalized by GAPDH with the correspondent densitometric analysis, * *p* < 0.05, ** *p* < 0.01, respect to sham. # *p* < 0.05 respect to BCNR; (**B**) Frozen section of the pelvic ganglia were immunostained with a polyclonal antibody against iNOS followed by a biotinylated secondary antibody and Vectastain^®^ ABC kit. 3,3'-Diaminobenzidine (DAB) was used as a chromogen and sections were counterstained with Hematoxylin. Magnification 400×. Bar= 50 μm.

The expression iNOS was further investigated at the protein level by Western blot (WB) and immunohistochemistry. Figure 4A shows the iNOS expression as determined by WB. We confirmed an up-regulation of iNOS by BCNR and a slight but statistically significant down regulation by SIL treatment. However, the level of iNOS measured for the SIL treatment group did not revert back to the measured levels of the sham operated control group. The histological localization of iNOS differs among groups. The expression of iNOS is observed in neurons and non-neurons cells in BCNR animals, although in SIL treatment group, the localization is only observed in glial cells (Figure 4B). The interaction of ROS and iNOS after BCNR was further confirmed by the increased expression of 3-nitrotyrosine, a marker of peroxynitrite formation which is a footprint of the interaction of anion superoxide and NO. The treatment with Sildenafil reduced the expression of 3-nitrotyrosine at a level similar to sham operated animals (not shown).

**Table 2:** Changes in the gene expression profile of nitric oxide and oxidative stress markers after BCNR and treatment with SIL determined by PCR array.

Symbol	Description	Sham *vs.* BCNR	BCNR *vs.* BCNR + SIL
Aass	Aminoadipate-semialdehyde synthase	−1.39	−3.19
Fos	FBJ osteosarcoma oncogene	2.22	−1.08
Gpx1	Glutathione peroxidase 1	1.89	−1.83
Gpx7	Glutathione peroxidase 7	2.08	−1.71
Mpo	Myeloperoxidase	1.16	−4.21
Ncf2	NADPH oxidase activator 2	2.22	−1.08
Nos2	Nitric oxide synthase 2, inducible	2.28	−1.96
Nos3	Nitric oxide synthase 3, endothelial cell	1.25	−1.05
Noxa1	NADPH oxidase activator 1	3.70	−4.62
p22-phox	Cytochrome b-245, alpha polypeptide	3.45	−2.26
Prdx6	Peroxiredoxin 6	1.54	−1.92

### 2.3. Expression of Heme Oxygenase 1 (HO-1) Parallels Inducible Nitric Oxide Synthase (iNOS) after BCNR and SIL Treatment

**Figure 5:**
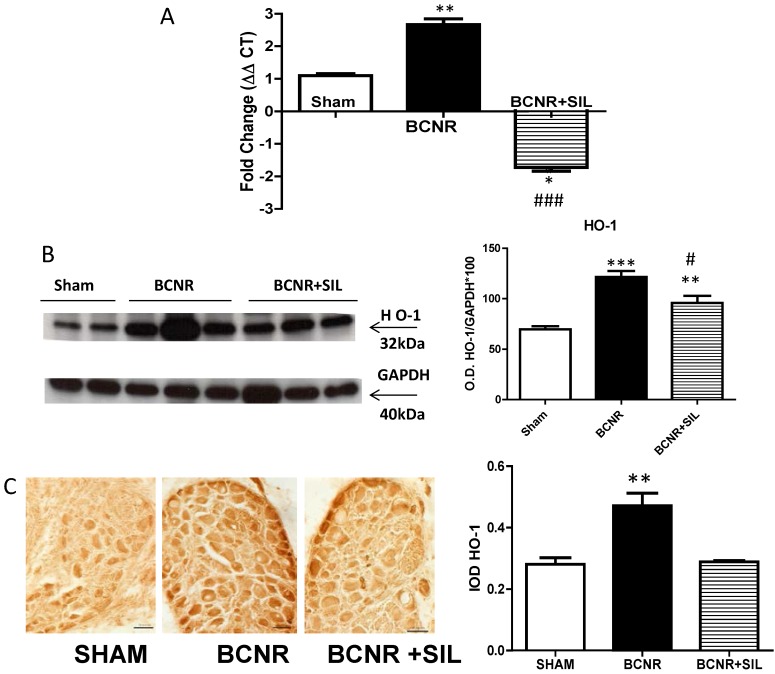
(**A**) Heme oxygyenase 1 is up-regulated by BCNR and down-regulated by Sildenafil treatment. Total RNA was extracted from 7 days Sham, BCNR and BCNR+ SIL and was subjected to real time PCR by TaqMan for HO-1 and normalized by the Ribosomal protein, large 1/3 (RPLP1/3) housekeeping gene; (**B**) Pelvic ganglia protein extracts were subjected to Western blot analysis for HO-1 by employing a monoclonal antibody normalized by GAPDH with the correspondent densitometric analysis; (**C**) Frozen pelvic ganglia sections from 7 days Sham, BCNR and BCNR+SIL were immunostained for HO-1 by employing a polyclonal antibody, with subsequent detection by DAB. No Counterstaining was performed. Representative pictures. Magnification 200×; Bar=50 µm. Image Analysis determined by integrated optical density (IOD) per cells in the pelvic ganglia *** *p* < 0.001; ** *p* < 0.01; * *p* < 0.05 with respect to Sham. # *p* < 0.05 , ### *p* < 0.001 with respect to BCNR.

It is well established that NO can activate heme oxygenase 1 (HO-1) gene expression and activity in a variety of tissues. However, like iNOS, HO-1 is also highly inducible by a vast array of stimuli, including oxidative stress, heat shock, ultraviolet radiation, ischemia-reperfusion (I/R), heavy metals, lipopolysaccharide, cytokines, NO and its substrate Heme. For that reason, we studied the HO-1 expression at both the mRNA level and protein level. As in iNOS expression, an up-regulation of the mRNA expression of HO-1 was observed after BCNR and was down-regulated by SIL treatment (Figure 5A). This was also confirmed at the protein level by Western blot and immunohistochemistry. BCNR induced an up regulation of the expression of HO-1, and a slight but significant down regulation was observed after SIL treatment measured by WB (Figure 5B) HO-1 expression by immunohistochemistry (Figure 5C) was primarily localized in neurons throughout the pelvic ganglia and was up regulated by BCNR and down-regulated by SIL treatment at levels comparable to Sham operated animals.

## 3. Discussion

The present study demonstrates that bilateral cavernosal nerve resection induces changes in the expression of oxidative stress markers and, inflammatory and anti-inflammatory cytokine regulation in major pelvic ganglia (MPG) neurons. The treatment with Sildenafil reduces oxidative stress by down-regulating the expression of NADPH oxidase, iNOS and HO-1. Even more, it also down-regulates the expression of inflammatory cytokines, such as IL-1β, IL-1Rα, and TNFα, whereas the levels of the anti-inflammatory or neuroprotective cytokines IL-6 and IL-10 remained elevated after 7 days of treatment with SIL.

Our results show, for the first time, an interaction between PDE5 inhibitors and cytokine modulation, indicating that these drugs have many other beneficial effects in regulating erectile function after nerve damage. In addition, we demonstrated that sildenafil treatment after BCNR results in stabilization of the oxidative stress level and prevention of inflammation in MPG neurons, which can result in amelioration of the neuropathic pain associated with nerve damage. These results are in agreement with previous findings showing that Sildenafil reduces oxidative stress and inflammatory injuries of the kidney in streptozotocin-diabetic rats [27] and after ischemia and reperfusion [28]. Expression of pro-inflammatory cytokines in injured nerves may have both beneficial and detrimental effects on nerve regeneration or neuropathic pain induction [29,30,31]. In the present manuscript, we demonstrated that BCNR up-regulates the expression of several pro-inflammatory cytokines in the pelvic ganglia; SIL treatment was able to reduce these levels back to that of sham operated animals. The exception was for IL-6 and IL-10 that were further up-regulated by SIL treatment. IL-6 and IL-10 are part of a group of cytokines that control the inflammatory response, in part, by regulating the synthesis and release of additional cytokines [11,32]. IL-10 acts mainly as an anti-inflammatory cytokine, whereas IL-6 is pro-inflammatory and regulates the growth, survival and differentiation of peripheral neurons [33]. Our results indicate that Sildenafil may exert an anti-inflammatory response by modulating the expression of inflammatory cytokines; possibly by interfering with multiple steps of leukocyte recruitment and migration [34,35] close to the injured neurons which may explain the reduction in the expression of MPO after SIL observed in our microarray results.

NADPH oxidase complex is known as the major source for the formation of ROS. The Nox family of NADPH oxidases is comprised of seven members, with tissue-specific expression patterns and different modes of action and function [36,37]. Our results indicate that BCNR induces the expression of important regulators and executive subunits of the NADPH oxidase such as Ncf2, p22-phox and Noxo1, which is indicative of increased superoxide production capacity as well as validated by the increased expression of nitrotyrosine, the footprint of superoxide–NO interaction. In addition, compensatory increases in antioxidant enzymes, GPX1 and GPX7 after BCNR were observed. Remarkably, all of the oxidative stress imbalances were stabilized by seven days of sildenafil treatment indicating that NO/cGMP pathway can modulate the activation of NADPH oxidase, the major enzyme involved in the generation of superoxide. These results are in agreement with previous findings demonstrating that SIL improves diabetic vascular activity through suppressing endothelin receptor A, iNOS, and NADPH oxidase [38], indicating a direct involvement of NO/cGMP pathway in the modulation of oxidative stress after BCNR in pelvic ganglia neurons.

Activation of iNOS in the nervous system plays an important role in mediating tissue damage through formation of peroxynitrites (OONO)—by a combination of ROS with NO and reducing NO bioavailability. Our results indicate that BCNR up regulates the expression iNOS and HO-1, two enzymes that are modulated by activation of NADPH oxidase-mediated superoxide [17,39], and the treatment with Sildenafil reduces the expression of those markers in the MPG. Further studies are needed to determine whether the effect of Sildenafil in down regulating iNOS, HO-1 and peroxynitrites formation is through the inhibition of NADPH oxidase or by a direct inhibition of iNOS expression and peroxynitrites formation.

In this study, we have not evaluated whether the attenuation of oxidative stress and cytokines modulation had an impact in promoting nerve regeneration, which is a limitation in the study. Although in our previous studies [26] we have demonstrated that Sildenafil treatment after BCNR promotes an up-regulation of neurotrophic factors, which strongly implies that Sildenafil may have an effect in promoting nerve regeneration. In line with our observations, the administration of plasma rich platelets injection in the corpus cavernosum, which contain various growth factors, increased the number of myelinated axons and facilitated recovery of erectile function in the bilateral cavernosal nerve injury rat model [40,41]. Further studies need to be done to confirm a direct involvement of the NO/cGMP pathway on nerve regeneration.

## 4. Experimental Section

### 4.1. Animals Treatment

Five-month-old male Fisher 344 rats (Harlan Sprague–Dawley, San Diego, CA, USA) were treated with an approved protocol by CDU Institutional Animal Care and Use Committee (protocol # I-1312-139, 1 December 2013), and divided as follows: (*n* = 8/group); Sham: sham-operated; BCNR: Bilateral cavernosal nerve resected; BCNR + SIL: BCNR + Sildenafil. BCNR was performed as we previously described [25,26,42,43]. In the sham-operated group both cavernosal nerves were identified but not resected. In the BCNR and BCNR + SIL groups, the main cavernosal nerves and ancillary branches were cut by removing a 5-mm segment. Sildenafil (Bayer, West Haven, CT, USA) was dissolved in drinking water (0.3 mg /mL), as previously described [25]. The drinking volume was measured daily and body weight was recorded weekly. The daily sildenafil dose (20 mg/kg body weight) was approximately equivalent to a single 200-mg tablet daily dose in men, when corrected for differences in total body surface area [25,44].

After the corresponding treatments, rats were killed by CO_2_ inhalation seven days after surgery. This time point was selected based on our previous studies of the histological and biochemical changes that occur in the penis start to become evident at 7 days after BCNR [4]. The pelvic ganglia (*n* = 8 animals per group) were dissected out and immersed in RNA later for RNA preservation (Ambion, Foster City, CA, USA).

### 4.2. RT^2^ Profiler™ PCR Array Analysis of Cytokines and Nitric Oxide Related Target Genes

For total RNAs, pelvic ganglia were pooled and isolated by homogenization with Trizol-Reagent (Invitrogen, Carlsbad, CA, USA). Total RNAs were purified by Qiagen Rneasy minicolumns (Qiagen, Valencia, CA) and stored at −80 °C until further used. RNA samples were subjected to reverse transcription, and the resulting cDNA was analyzed by a customized rat oxidative stress and cytokines RT^2^Profiler™ PCR Array (SA Biosciences Corp., Frederick, MD, USA), which contains a panel of 84 primer sets of genes related cytokines, oxidative stress and Nitric oxide signaling pathway. Real-time PCRs were performed as follows: melting for 10 min at 95 °C, 40 cycles of two-step PCR including melting for 15 s at 95 °C, annealing for 1 min at 60 °C. The raw data were analyzed using the ΔΔ*C*_t_ method following the manufacturer’s instructions (SA Biosciences Corp., Frederick, MD, USA) [25,26,45]. Only genes that expressed above ±1.8 fold changes were considered significant with its respective controls.

### 4.3. Heme Oxygenase Determination by TaqMan^®^ Real Time PCR

The levels of mRNA expression of heme oxygenase 1 (HO-1) in the pelvic ganglia of sham, BCNR and BCNR + SIL were measured by quantitative real-time PCR using custom TaqMan^®^ primers and FAM labelled TaqMan^®^ Minor Groove Binder (MGB) probes (Applied Biosystems, Life Technologies, Grand Island, NY, USA). Real-time PCR reactions were set up by combining 10 μL TaqMan^®^ Universal Master Mix II no UNG (Applied Biosystems), 1 μL of forward and reverse primers (previously diluted to 10 μM), and 9 μL of 50 ng/μL life-cycle and isolate-specific cDNA to give a total volume of 20 μL. Triplicate reactions were set up for each sample, isolate and life-cycle stage combination in a MicroAmp™ Optical 96-well plate sealed with a MicroAmp™ Optical adhesive film (Applied Biosystems). All real-time PCR runs were carried out on a StepOnePlus system (Applied Biosystems), using the following conditions: 95 °C for 10 min followed by 40 cycles of 95 °C for 15 s and 60 °C for 60 s. Collection of data occurred during the final phase of each cycle. Triplicate real-time PCR runs were carried out for each gene under test. The cycle threshold (*C*_t_) values were recalculated by setting the baseline and threshold to identical values for each of the three runs to enable direct comparison among the runs. Relative quantification of the gene expression level was carried out using the comparative *C*_t_ (ΔΔ*C*_t_) method [26,45,46] and determined as the difference between the *C*_t_ for a specific mRNA gene and the *C*_t_ for a reference mRNA, normalized to RPlP1,3 threshold expression.

### 4.4. Proteome Array for Determination of Cytokines and Chemokines

To determine the changes of the cytokine profile in pelvic ganglia after BCNR and SIL treatment, the rat cytokine proteins array (R&D System Inc., Minneapolis, MN, USA) was employed following the manufacturer instructions. Briefly, protein aliquots of 200 µg were diluted and mixed with a cocktail of biotinylated detection antibodies for 1 h at room temperature. The sample/antibody mixture was then incubated overnight at 4 °C with the membrane array. The cytokine/detection antibodies complex present were bound by its cognate immobilized capture antibodies on a nitrocellulose membrane containing 29 different anti-cytokine antibodies in duplicate. After several washes, streptavidin-horseradish peroxidase was added and incubated for 30 min. Membranes were then exposed to Western blot chemiluminescent detection reagents (GE Healthcare, Piscataway, NJ, USA). The densitometric signal produced by each spot run in duplicate was proportional to the amount of cytokine bound as determined by Image J (National Institute of Health Bethesda, MD, USA). Positive and negative controls are included in each membrane array to compensate for background and intensity differences [46].

### 4.5. Western Blot Analysis

Pelvic ganglia proteins were extracted from Trizol fractions according to the manufacturer instructions. Protein aliquots (30–50 μg) were subjected to Western blot analyses [26] by 4%–15% Tris-HCl polyacrylamide gel electrophoresis (PAGE) (Bio-Rad, Hercules, CA, USA) in running buffer (Tris/Glycine/SDS). Proteins were transferred for 3 h to nitrocellulose membranes in transfer buffer (Tris/glycine/methanol). Afterward, the non-specific binding was blocked by immersing the membranes into 5% non-fat dried milk and 0.1% (*v*/*v*) Tween-20 in PBS (1X) overnight at 4 °C. After several washes with washing buffer (PBS Tween 0.1%), the membranes were incubated with the primary antibodies for 3 h at room temperature as follows: (**a**) a rabbit polyclonal IL-1 B (1:500) (Santa Cruz, Cambridge, MA, USA); (**b**) a polyclonal antibody iNOS (1:500) (BD Bioscience, San Jose, CA, USA); (**c**) monoclonal antibody HO-1 (Chemicon International, Temecula, CA, USA); (**d**) Glyceraldheyde-3-phosphate dehydrogenase (GAPDH) (1/10,000) (Chemicon International). The washed membranes were incubated for 1.5 h at room temperature with 1/2000 dilution of secondary antibodies, anti-mouse or anti-rabbit respectively, linked to horseradish peroxidase. After several washes, the immunoreactive bands were visualized using the ECL plus Western blotting chemiluminescence detection system (Amersham Biosciences, Piscataway, NJ, USA). The densitometric analysis of the bands was performed with Image J (NIH, Bethesda, MD, USA). A positive control was run in all gels for each antibody to standardize for variations in exposures and staining intensities. Negative controls were performed by omitting the primary antibody. Band intensities were determined by densitometry and the respective intensities were corrected by GAPDH, a housekeeping protein, upon re-probing the same membrane.

### 4.6. Retrograde Labeling of Pelvic Ganglion Cells

Pelvic ganglia neurons that specifically innervate the penis were traced by pressure injection of the fluorescent tracer fluorogold (FG, Fluorochrome Inc., Engelwood, CO, USA), to the penis, one week before any experimental manipulation. Briefly, under general isoflurane anesthesia, rats were injected 7 days before sham or BCNR surgeries with 4% FG into the cavernosal space of the penis at multiple sites (2 μL each, maximum 8–10 µL) using a 10-µL Hamilton syringe and a 30-gauge needle. Overflow of tracer solutions were rinsed with sterile saline, and the injection sites were dried and coated with Dermabond to prevent the spread of dye [26]. To demonstrate the specificity of the retrograde tracing, a group of animals were injected with FG into the peritoneal cavity as negative controls. Animals were sacrificed 14 days after FG injection to allow the dye to reach the spinal cord.

After treatments, rats were deeply anesthetized with pentobarbital (100 mg/kg body weight), perfused with saline followed by 4% *p*-formaldehyde (PFA). The pelvic ganglia were dissected out, post-fixed in 4% PFA overnight and transferred to 25% sucrose until sectioning.

### 4.7. Immunohistochemistry and Immunofluorescence

The detection of iNOS and HO-1 by immunohistochemistry was carried out on 16 µm frozen sections [26]. Sections were washed with PBS, quenched for endogenous peroxidase activity, blocked with 10% normal goat serum in PBS in 0.15% triton X-100, and incubated overnight in a humidified chamber at 4 °C with the following primary antibodies: anti-iNOS (1/400) (BD Biosciences, San Jose, CA, USA), anti-HO-1 (1/250) (ABCam, Cambridge, MA, USA). Biotinylated goat anti-rabbit or horse anti-mouse secondary antibody (1/200) (Vector Laboratories, Burlingame, CA, USA) were applied and incubated for 40 min, followed by 30 min with ABC peroxidase complex (Vector Laboratories, Burlingame, CA, USA) and diaminobenzidine as a chromogen. Sections were dehydrated and cover slips mounted. Negative controls were done by replacing the first antibody with non-immune IgG. Each slide assayed had a negative control. Sections were counterstained with hematoxylin except for HO-1 immunostaining.

### 4.8. Co-Localization of Fluorogold Positive Neurons with IL-1β and IL-6 by Immunofluorescence

Frozen sections were pre-incubated with 10% goat serum or horse serum and later on with a 1/500 dilution of the anti IL-1β polyclonal antibody, or 1/500 dilution of IL-6 antibody followed by a 1/200 dilution of anti-rabbit biotinylated secondary antibody (Vector Laboratories, Burlingame, CA, USA) respectively. Sections were then incubated in 15 mg/mL of streptavidin-Texas red (TR); (Vector Laboratories, Burlingame, CA, USA). Sections were mounted in DAPI Vectabond [26] (Vector Labs.) and examined under an Olympus microscope. For fluorogold visualization a wide UV filter was used.

### 4.9. Quantitative Image Analysis

Quantitative image analysis (QIA) was performed using the ImagePro Plus 7.0 program (Media Cybernetics, Silver Spring, MD, USA) [25,26,42,43,44,45,46]. For the determination of HO-1 and iNOS, the total integrated optical density (IOD) was used to determine changes in the expression. After the images were calibrated for background lighting, IOD was proportional to the concentration of antigen assayed. In all cases, at least 4 matched sections per animal and 8 animals per group were analyzed in each of the groups [4,25,26,42,43,44,45,46].

### 4.10. Statistical Analysis

Values were expressed as Mean ± SEM. The normality distribution of the data was established using the Wilk–Shapiro test. Multiple comparisons were analyzed by a one-way analysis of variance (one-way ANOVA) followed by *post hoc* comparisons with the Tukey test, using GraphPad Prism v5.0. Differences were considered significant at *p* < 0.05.

## 5. Conclusions

Cavernosal nerve damage promotes activation of pro- and anti-inflammatory cytokines together with an up-regulation of oxidative stress markers in MPG neurons. The treatment with sildenafil resulted in stabilization of the oxidative stress level and prevention of inflammation in MPG neurons by modulating cytokines expression and promoting a neuroprotective environment favoring neuron survival after nerve damage. In addition, initiation of the treatment right after surgery or even before the surgery will produce a better outcome in promoting attenuation of the inflammatory response and activation of neurotrophic factors that will hence promote nerve regeneration. This data encourages further evaluation of PDE5 inhibitors as anti-inflammatory and neuroprotective agents.

## Abbreviations

SIL: Sildenafil
BCNR: bilateral cavernosal nerve resection
iNOS: inducible nitric oxide synthase
HO-1: Heme oxygenase 1
IL-1: Interleukin 1
IL-6: Interleukin 6
RPlP1,3: Ribosomal protein, large 1/3
GAPDH: glyceraldehyde-3-phosphate-dehydrogenase
IMM: immunohistochemistry
